# Effect of Sulfur on Wood Tar Biopitch as a Sustainable Replacement for Coal Tar Pitch Binders

**DOI:** 10.1021/acsaenm.3c00361

**Published:** 2023-10-06

**Authors:** Zeban Shah, Mohammadhossein Saberian, Darren Hodgeman, Ian Kinloch, Cristina Vallés

**Affiliations:** † Department of Materials, National Graphene Institute and Henry Royce Institute, University of Manchester, Oxford Road, Manchester M13 9PL, U.K.; ‡ Carbon Science Center of Excellence, Morgan Advanced Materials and Technology, Inc., 310 Innovation Boulevard, Technology Center, Suite 250, University Park, Pennsylvania 16803, United States

**Keywords:** coal tar pitch, wood tar biopitch, carbon−carbon composites, graphitization promotor, electrical properties, mechanical properties

## Abstract

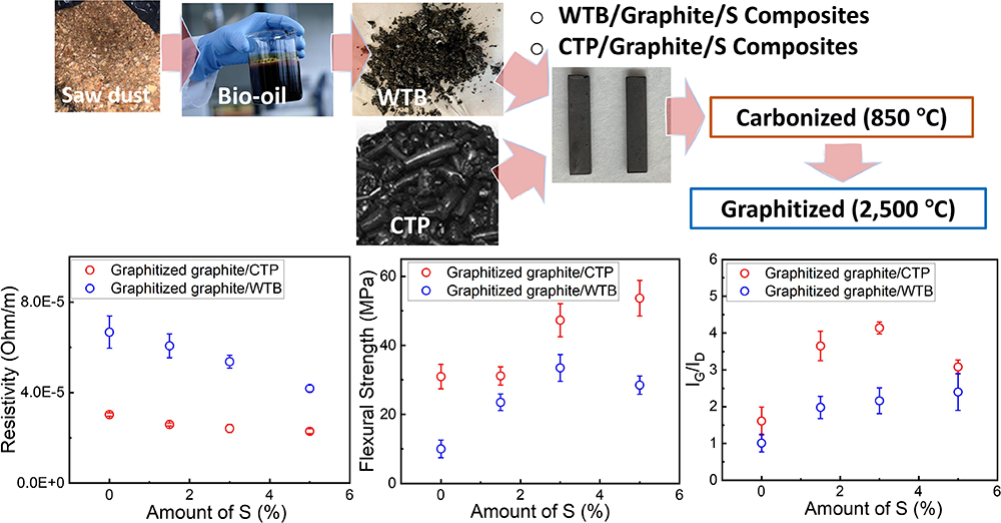

Coal tar pitch (CTP) is a residue formed from the distillation of coal tar and is widely used as a carbonizable and graphitizable binder for many industrial applications. However, CTP is fossil-derived and has recently been classified as a “sunset” status material under REACH due to its toxicity, which makes finding a sustainable alternative vital. In this work, bio-oil was synthesized from the pyrolysis of fresh eucalyptus sawdust, from which wood tar biopitch (WTB) was subsequently produced by a second distillation process. Chemical characterization revealed the presence of higher amounts of aromatic compounds and PAHs in the industrially used CTP relative to the WTB. Sulfur is widely used as a graphitization promoter for CTP but has not yet been used for biopitch alternatives. Hence, graphite/WTB and graphite/CTP composites were fabricated with varying amounts of sulfur and were subsequently carbonized and graphitized at 850 and 2500 °C, respectively. The use of WTB as a binder led to less porous composites after carbonization/graphitization with higher levels of shrinkage than those based on CTP, whereas the carbon yield was very similar for both systems. The incorporation of sulfur was found to promote more compact structures with higher levels of graphitization, leading to improved electrical and mechanical properties, particularly for the composites based on CTP due to the higher levels of graphitization achieved relative to the WTB. The electrical and mechanical performance found for the WTB-based composites, combined with the much lower toxicity, evidences the promise of WTB as a sustainable alternative to traditional CTP binders.

## 1 Introduction

Coal tar pitch (CTP) is a residue formed from the distillation of coal tar and is widely used as a carbonizable/graphitizable binder to form carbon electrodes (e.g., for aluminum smelting), seals, specialty graphites for electric brushes and current collectors (e.g., wind turbine generators and rail pantograph systems), and molten metal-conveying components for the metal industries. Due to its good binding properties, high mixability, and strong interaction with carbon particles, such as coke, carbon black, and graphite, as well as its ability to penetrate their open pores transforming these mixtures of carbon particles into moldable pastes, CTP is highly used as a carbonizable/graphitizable binder for many applications in the Foundation Industries.^1−3^ Typically, carbon–carbon composites are produced by binding carbon particles with CTP and then carbonizing and graphitizing them at temperatures above 2000 °C to give high electrical and mechanical properties. In demanding applications, a high degree of graphitization is essential, making optimization of the processing and the structure of the systems very important. CTP, however, is fossil-derived and has recently been classified as a “sunset” status material under REACH due to its carcinogenicity. In addition, increasing environmental regulations are currently raising serious concerns about the long-term availability and supply of CTP. Thus, it is vital to identify sustainable alternative binders to CTP that reduce the health and environmental issues while keeping the properties required by the Foundation Industries who rely on these materials.^4−7^ Furthermore, the products made with CTP wear in service and cannot be easily recycled, making a bioderived material the only viable solution.

In the last decades, there has been increasing interest in the synthesis of biomass as a clean source of fuel due to its renewability and sustainability. Around 30 years ago, when new technologies made the conversion of biomass feedstock into valuable products feasible, researchers started working on the development of new concepts and processes for the thermochemical conversion of biomass into biopitches. Already in the 90s, eucalyptus wood-derived cokes and pitches were successfully synthesized at a bench scale and were mixed together to fabricate electrodes, with the electrode grade carbons prepared with bio-derived and petroleum-derived carbon materials showing comparable physical properties.^8,9^ Later, the same research group reported that the electrical conductivity, mechanical properties, and thermal expansion of their biocarbon-based electrodes were also comparable to those found for electrodes based on a fossil-derived pitch.^3^ However, despite an enormous academic interest, socio-economic drivers have only recently become sufficient to address key shortcomings associated with the synthesis of wood tar biopitch (WTB) and take it forward. This has renewed the interest in synthesizing wood-derived carbonizable/graphitizable materials as alternative binders to the fossil-derived ones (i.e., CTP) for industrial use, and recent works can be found in the literature. For example, the synthesis of WTB from bio-oil produced from a mixture of pine and cedar sawdust has been recently reported and it was found to contain much lower PAHs, quinoline insolubles, and sulfur contents than CTP, showing significant health and environmental advantages as a binder. The effect of the synthesis conditions on the WTB properties was also evaluated, finding that higher distillation times and temperatures led to lower yields of biopitch but with higher C/H ratio and aromaticity. It was also shown that some distillation parameters, such as temperature, heating rate, and pressure, can be controlled to adjust the softening point and the viscosity of the synthesized biopitch, which is relevant for the fabrication of anodes.^5^ WTB has also been synthesized through the thermal degradation of coniferous and deciduous tree sawdust. These WTBs showed a lower coking value and quinoline-insoluble matter in comparison to CTP, also leading to the production of significantly reduced emissions of PAHs. The carbon composite samples manufactured with these wood-derived binders mixed with coke and graphite particles showed similar density and mechanical compression strengths compared to those based on the conventional CTP after carbonization at 1000 °C and graphitization up to 2800 °C.^10^ In a different work, carbon composites prepared mixing biopitch with coke particles followed by carbonization at 1100 °C were found to achieve similar density, coefficient of thermal expansion, specific electrical resistivity, mechanical strength, and lower air permeability relative to the carbonized composites based on CTP. However, the WTB samples possessed a more amorphous microstructure, as well as higher air reactivity and specific electrical resistivity.^11^ All these reported wood-derived alternative carbon binders show promise as more sustainable CTP replacements, due to their important health and environmental advantages, good mixing, wetting, and binding with solid carbon particles to effectively form carbon–carbon composites, as well as the feasibility to control their C/H ratio and aromaticity, carbon yield, carbonization/graphitization degree, softening point, and viscosity by varying the experimental conditions employed for its synthesis, which will allow a control of the electrical and mechanical properties of their resultant carbonized/graphitized composites. However, the overall properties found so far for the composites containing WTB as binder are still relatively poor relative to those found for CTP-based composites due to its more amorphous microstructure. Elemental sulfur (S) has been widely used in the carbon industry as a promotor to improve the structure and, thus, the properties of the carbon composites based on CTP. However, to our best knowledge, no one has yet investigated the effect of S on the structure and properties of these recently developed wood-derived binders. Hence, there is no literature or theoretical knowledge at the moment to support a definitive mechanism on how sulfur promotes the graphitization of such carbon composites.

Herein, bio-oil was successfully synthesized from the pyrolysis of fresh eucalyptus sawdust and WTB was subsequently produced from the bio-oil following a second distillation process. The chemical composition and content of aromatic compounds and PAHs of the synthesized WTB were analyzed and compared to those found in the industrially used CTP in order to evaluate the potential of WTB as a more sustainable carbonizable/graphitizable binder. Both WTB and CTP binders were mixed with graphite particles to fabricate a model composite system with different amounts of S incorporated as a promotor. These mixtures were then carbonized and graphitized to produce carbon–carbon composites. The microstructure, carbon yield, open porosity, density, and shrinkage, as well as the electrical and mechanical properties of the composites, were evaluated and compared against each other. The structural changes occurring during carbonization and graphitization were investigated, with particular focus on understanding how the presence of S affected these changes and, thus, the composite properties. This comparison between the structure and performance of the two carbon binders incorporating the industrial standard promotor S is key to assessing the usability of WTB as a more sustainable binder in applications such as metallurgy conveying equipment, mechanical seals, and electrical components, as well as to identifying the remaining technical and processing challenges for industrial uptake of WTB. Ultimately, these sustainable products hold great potential to filter downstream into other industries (e.g., electrified rail and wind turbine sectors) and contribute to the wider transition in other foundational sectors such as aluminum smelting, which is the largest user of CTP.

## 2 Experimental Section

### 2.1 Materials

Fresh eucalyptus sawdust with an average particle size of ∼500 μm was used as the starting material for the synthesis of WTB. CTP and natural graphite powder with particle lateral dimensions of ∼17 μm were provided by Morgan Advanced Materials. Elemental S with a purity of 99.5% was purchased from Thermo Fisher Scientific.

### 2.2 Production and Characterization of Bio-Oil

**Figure 1:**
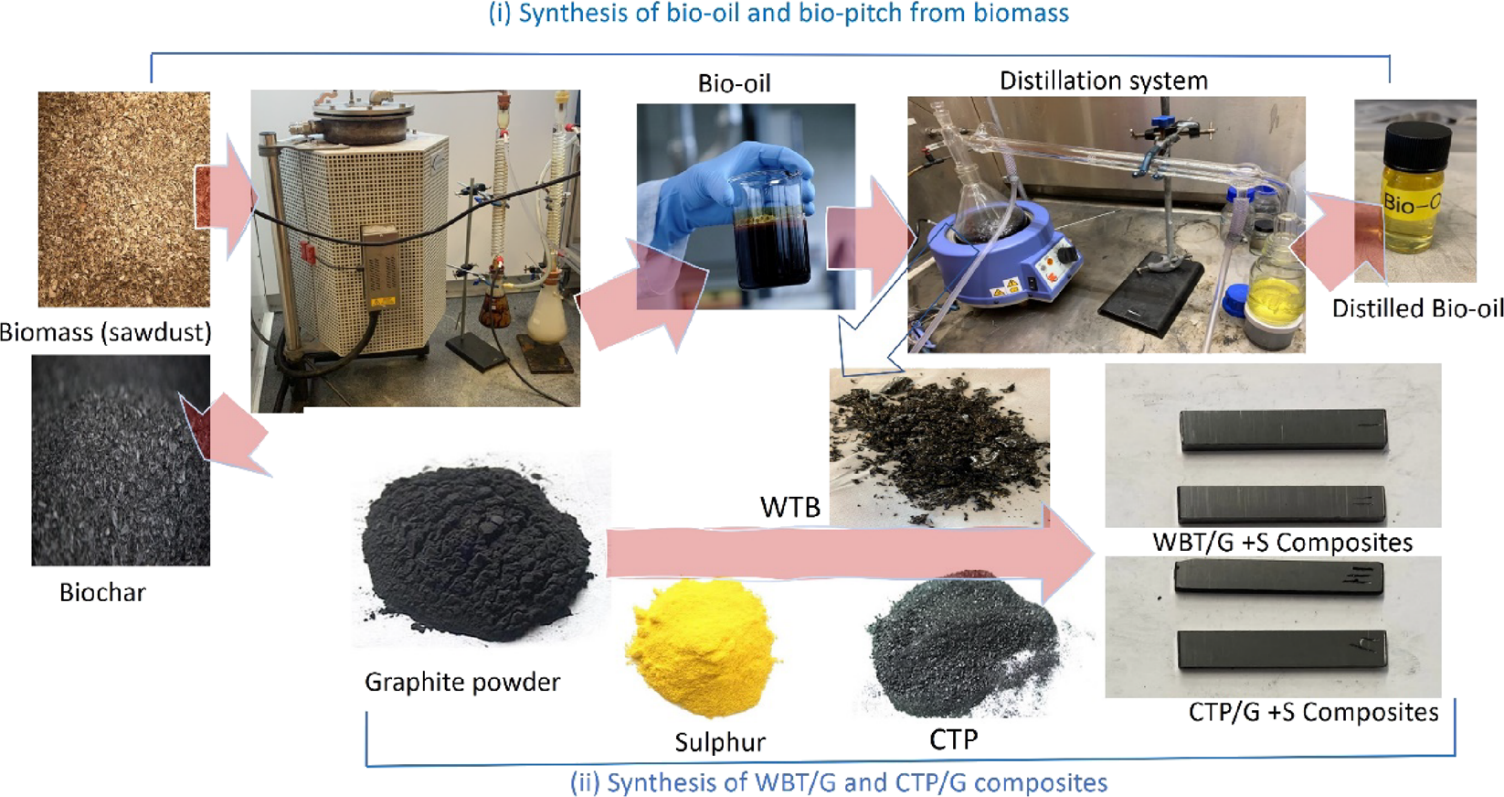
Schematic of the pyrolysis/distillation process to produce bio-oil from sawdust and further distillation process to synthesize WTB. Pictures of WTB, CTP, graphite powder, sulfur powder, and graphite/WTB and graphite/CTP composites are shown.

Bio-oil was synthesized from the pyrolysis of fresh eucalyptus sawdust under an Ar atmosphere using an in-house pyrolysis system following a method previously reported^12,13^ and is schematically shown in Figure 1. 1 kg of eucalyptus sawdust was introduced in a stainless-steel reactor, which was connected to two condensers placed in series. The reactor was heated to 900 °C at a rate of 10 °C/min and kept at this reaction temperature for 1.5 h. During that time, the biomass was converted to biochar (i.e., the solid residue from its pyrolysis) while biogas was released and condensed in the two condensers, which were kept at a temperature of 7 °C. The condensed biogas comprised a mixture of water and bio-oil, and the bio-oil fraction was separated from the water using a separatory funnel. Due to their different densities, after 2–3 h, two clearly separated layers were formed, with the bio-oil layer being on top of the water fraction. 120 g of bio-oil was typically obtained from 1 kg of sawdust, which corresponds to a yield of ∼12% (it should be noted that this low yield may be a result of focusing on biopitch quality rather than bio-oil quantity). The synthesized bio-oil was characterized by thermogravimetric analysis (TGA) and Fourier transform infrared (FTIR) spectroscopy using a Mettler-Toledo DSC/TGA analyzer and a Nicolet iS50 FTIR spectrometer, respectively, and the results are shown in the SI.

### 2.3 Production and Characterization of WTB

WTB was produced by distilling the synthesized bio-oil (Figure 1). 300 mL of bio-oil was introduced in an atmospheric distillation system and heated from room temperature to 150 °C at a heating rate of 3 °C/min and then held until 50% of the initial mass of bio-oil was evaporated (i.e., until 150 mL of the starting bio-oil was evaporated, which was controlled using a graduated beaker). The remaining dark viscous material was left to cool to room temperature and was labeled as “WTB”, whereas the condensed evaporated fraction was labeled as “distilled bio-oil” (Figure 1). The FTIR spectrum of the WTB was obtained using a Nicolet iS50 FTIR spectrometer in the frequency range of 4000–400 cm^–1^, and its TGA was performed using a Mettler-Toledo DSC/TGA analyzer up to 1000 °C under a N_2_ atmosphere using a heating rate of 10 °C/min and a flow rate of 100 mL/min. The CTP provided by Morgan Advanced Materials was characterized using identical procedures and was used as a reference.

The coking values (CVs) of the WTB and CTP were obtained by a modified Conradson method using the standard ASTM D2416-20. Three grams of sample was placed in a furnace and heated up to 900 °C under an Ar atmosphere followed by a 30 min dwell, after which it was removed and cooled down to room temperature. The CV was calculated from the residual mass using eq 1:
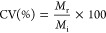
1where *M*_i_ is the initial mass of the sample and *M*_r_ is the mass of the residue (i.e., the mass of the sample after the thermal treatment applied).

The polycyclic aromatic hydrocarbon (PAH) content of WTB and CTP was determined by the U.S. Environmental Protection Agency (EPA) Method 8270D for semivolatile organic compounds by Applied Technical Services (ATS). Due to its toxicity, relevant safety precautions were implemented in the lab to manipulate the CTP, which includes wearing appropriate PPE, working in a fumehood, and using suncream to avoid potential skin burns.

### 2.4 Fabrication and Characterization of Carbon–Carbon Composites

Graphite/WTB and graphite/CTP (50/50 by weight) carbon–carbon composites were fabricated by mixing the binder with natural graphite powder in a blender (Ika Werke M20) at room temperature for 10–15 min. The resultant mixture was heated to 120 °C for 3 h to evaporate any low molecular weight volatiles. Graphite/binder green composites were produced by pressing the mixture into a 40 mm × 10 mm × 4 mm mold under a pressure of 61 MPa for 5 min. These green composites were subsequently carbonized under an Ar atmosphere at 850 °C using a heating rate of 35 °C/h and a dwell time of 30 min to produce carbonized graphite/binder composites, which were subsequently graphitized at 2500 °C under a vacuum using a heating rate of ∼25 °C/min and a dwell time of 30 min, leading to graphitized graphite/binder composites. Three repeat samples were produced for each mixture and set of conditions studied.

With the objective of evaluating the role of S as a promoter in these carbon–carbon composites, different amounts of elemental S (1.5, 3, and 5 wt % by weight relative to the binder) were added to the system during the mixing process and composites were then produced as described above.

The apparent density (g/cm^3^) and open porosity (%) of the green, carbonized, and graphitized composites fabricated were determined by hydrostatic weighting (i.e., Archimedes' principle). The % of shrinkage occurring during the carbonization and graphitization steps were calculated using eq 2:
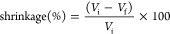
2where *V*_*i*_ and *V*_*f*_ are the initial and final volume of the specimens, respectively.

The carbon yield (%) of the carbonized and graphitized composite samples was calculated using eq 3:
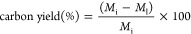
3where *M*_i_ is the initial mass of the binder used during the mixing process and *M*_l_ is the mass loss occurring during carbonization/graphitization (the graphite filler was found not to change mass).

The microstructures of the carbonized and graphitized samples were characterized by scanning electron microscopy (SEM) using a Quanta 250 SEM operating at 20 kV. Raman spectra of the composites were recorded using a Renishaw 1000 Raman spectrometer equipped with a 633 nm He–Ne laser. To prevent laser-induced heating, a low laser power (∼10 mW) was used. The elemental analysis of the green, carbonized, and graphitized composites was performed using an organic elemental analyzer Thermo FLASH 2000.

The electrical resistance of the carbonized and graphitized composites was determined by the four-point probe method using a Keithley Tektronix 2450 SourceMeter. Three-point bending tests were performed on the graphitized composites using an Instron 3365 Universal Testing System to determine their flexural strength and moduli. These tests were performed on 40 mm × 10 mm × 4 mm specimens using a crosshead speed of 1.5 mm/min.

## 3 Results and Discussion

### 3.1 Chemical Composition and PAH Content in WTB and CTP

**Figure 2:**
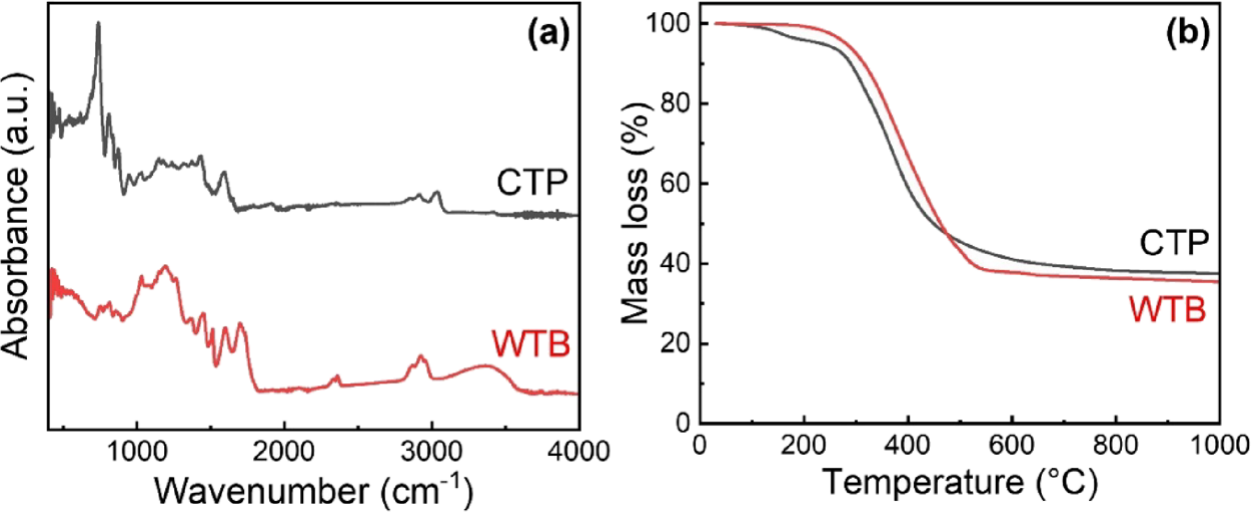
FTIR (a) and TGA (b) images of WTB and CTP.

The chemical composition of the WTB synthesized in our lab and the industrially used CTP were characterized by FTIR, and the results are shown in Figure 2a. For clarity, a table summarizing the information revealed by these FTIR spectra is also included in the SI. These data show that both WTB and CTP binders show vibrations in the 3300–3600 cm^–1^ region, which are typically associated with the presence of moisture, hydrogen bonds, amines, and the C–O stretch of −COOH groups. C–H stretching vibrations found in the range 2835–2940 cm^–1^ and features appearing in the 1440–1460 cm^–1^ region could be also identified in both spectra, which are associated with the vibrations of CH_2_ and CH_3_ groups of aliphatic groups in both binders. The peaks appearing in the region between 1110 and 1270 cm^–1^ also reveal that both materials contain aliphatic hydrocarbons and oxygen-containing nonaromatic chemical compounds, such as aliphatic ethers, phenols, and alkyl aryl ethers. Even though the presence of all these chemical groups can be identified in the FTIR spectra of both binders, they appear in considerably different amounts. The higher relative intensity of the bands associated with the vibrations of all these functional groups found for the WTB suggests that this pitch must contain higher amounts of all of them relative to CTP. In addition, the FTIR spectrum of the WTB shows some vibrations in the range of 1700–1740 cm^–1^, which are typically related to C=O stretching in ketones (1700–1725 cm^–1^) and aldehydes (1720–1740 cm^–1^), indicating that this binder has these C=O-containing chemical compounds, whereas they could not be found in the CTP. Finally, another important difference between their chemical composition is the presence of very intense peaks between 600 and 860 cm^–1^ in the CTP spectrum, which correspond to the C–H stretching of aromatic groups and are not present in the WTB spectrum. The FTIR spectra of these two binders clearly show different chemical compositions, which must be attributed to the very different routes followed for their synthesis, with the CTP being derived from coal tar (widely known to be a highly toxic and environmentally hazardous material due to the presence of a high number of aromatic molecules in its structure)^3,14,15^ and the WTB being synthesized from biomass.

The aromaticity of these materials represents an important parameter when they are evaluated as industrial binders. Indeed, their aromaticity can be considered a measure of graphitizability, which will determine the final structure of their carbonized/graphitized carbon composites and thus their properties. In addition, it provides information about their health and environmental advantages/disadvantages, which will determine whether they are viable as sustainable binders. Thus, in order to get a deeper insight into the aromaticity of the WTB relative to the CTP, a qualitative and quantitative evaluation of the PAHs present in both binders was performed using the EPA method. The data obtained (SI) revealed that less PAHs and at considerably lower concentrations were found in the WTB synthesized in our laboratories relative to the CTP used here. In particular, the WTB was found not to contain benzo[*a*]pyrene, which is considered one of the most hazardous and carcinogenic PAHs typically found in coal tar-derived pitches. It is worth mentioning that, for safety reasons, the CTP used in this work has gone through an industrial washing process before being delivered to us in order to remove the chemicals, which have been classified as “sunset” under REACH, such as the benzo[*a*]pyrene. Thus, the CTP used here was found not to contain that particular PAH either (SI) whereas the CTP typically used in industry (i.e., nonwashed) contains benzo[*a*]pyrene at concentrations >10,000 mg/kg.^16^ In addition to this highly carcinogenic PAH, the nonwashed CTP must contain higher amounts of PAHs than the CTP used in this work, making the difference in aromaticity between CTP and WTB even more evident. The WTB synthesized in our laboratories from fresh sawdust seems, thus, to represent a more sustainable and environmentally friendly binder than the CTP currently used in industry. However, this lower aromaticity found for the WTB also suggests that this bioderived pitch must be less graphitizable than the CTP, which might have a negative impact on the structure and properties of its carbonized/graphitized composites.

### 3.2 CV and Thermal Behavior of WTB and CTP

The CV is typically defined as the amount of solid coke (i.e., carbon-based material) remaining after a pitch is heated to 900 °C under an inert atmosphere and is a very important parameter in determining whether a particular binder is economically viable. When these binders are heated up to 900 °C under an inert atmosphere, the functionalities and PAHs present in their structures will be thermally degraded, leaving a material composed predominantly of atoms of carbon, which will be subsequently graphitized to render highly ordered carbon structures. CVs of ∼34 and ∼46 wt % were found for the WTB and the CTP, respectively, using a modified Conradson method. The different values found for both binders must be related to their different chemical compositions (revealed by FTIR and the EPA method), with the different chemical compounds and volatiles present in them being released at different temperatures and rates during the thermal treatment.

In order to investigate this further, the nonoxidative thermal stability of both materials was evaluated by TGA (Figure 2b). Both materials show their main mass loss between 250 and 500 °C (∼57 and ∼50 wt % for WTB and CTP, respectively). The curve obtained for the CTP showed a small weight loss peak of ∼4 wt % at ∼150 °C with the main weight loss at ∼360 °C, whereas for the WTB, only one main weight loss could be observed at ∼380 °C. The small weight loss found for the CTP at ∼150 °C is assumed to be due to the low molecular weight components (e.g., butene and pentene),^17,18^ which are not present in the WTB (they have been removed during the distillation followed for its synthesis), whereas the WTB contained higher molecular weight compounds (e.g.*,* tridecane and hexadecane), which need slightly higher temperatures to decompose. Above 500 °C, a progressive weight loss was still observed in both samples leaving final carbon residues of ∼35 and ∼37 wt % at 1000 °C, in the case of WTB and CTP, respectively. These residual masses determined by TGA differ slightly from the CVs obtained by the Conradson method, which we attribute to the different conditions employed during both heating processes (i.e., inert atmosphere, heating rates, and dwelling times). Both techniques led, however, to a slightly higher residual mass for the CTP relative to the WTB, with this difference being relatively small, which highlights the promise of WTB as a binder.

### 3.3 Carbon–Carbon Composites Based on WTB and CTP

#### 3.3.1 Microstructure of the Carbonized and Graphitized Composites

**Figure 3:**
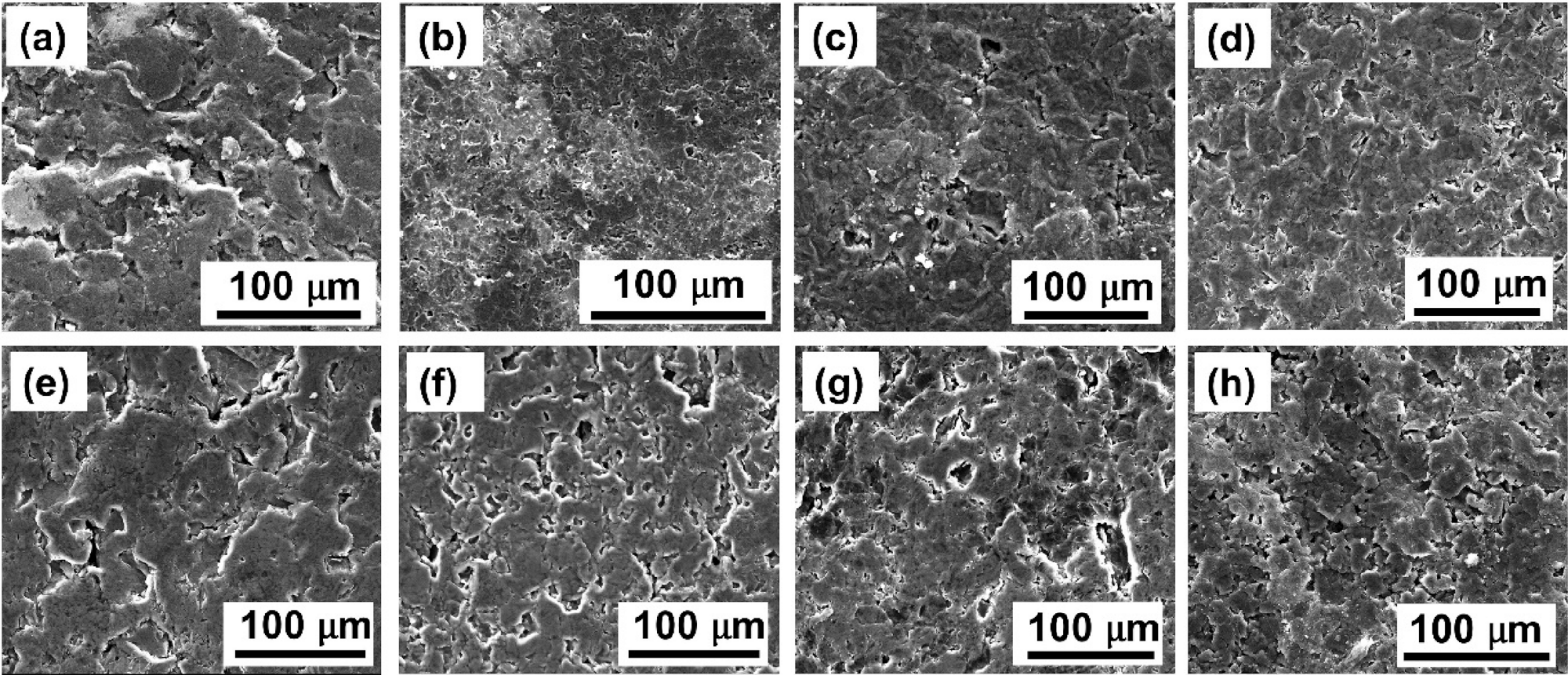
SEM images of carbonized graphite/WTB (a), graphitized graphite/WTB (b), carbonized graphite/WTB/S (5 wt %) (c), graphitized graphite/WTB/S (5 wt %) (d), carbonized graphite/CTP (e), graphitized graphite/CTP (f), carbonized graphite/CTP/S(5 wt %) (g), and graphitized graphite/CTP/S (5 wt %) (h) composites.

The microstructures of the carbonized and graphitized composites based on CTP and WTB fabricated with different contents of S were characterized by SEM, and representative images of their surfaces are shown in Figure 3. These SEM images reveal compact structures with no voids, large pores, or cracks in all the cases, which is further supported by SEM images of their cross sections (SI). The observed microstructures imply good binding of both the CTP and WTB to the graphite particles. No clear structural differences could be found between carbonized and graphitized composites, and the addition of S did not give any noticeable structural differences.

**Figure 4:**
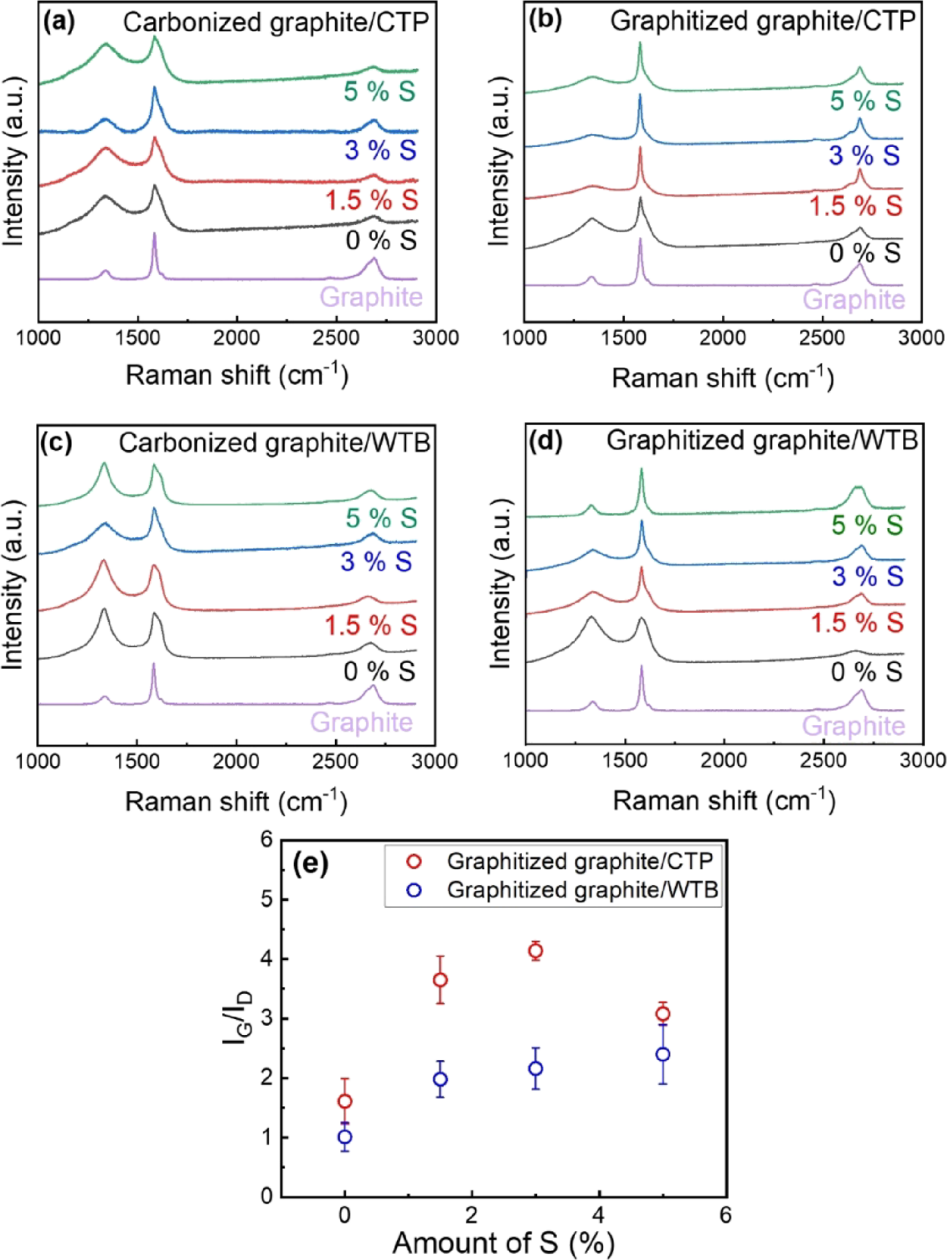
Raman spectra of carbonized (a) and graphitized (b) graphite/CTP composites and carbonized (c) and graphitized (d) graphite/WTB composites with different S contents (the Raman spectrum of the graphite is also shown as a reference). Variation of *I*_G_/*I*_D_ with the S content for the graphitized graphite/CTP and graphite/WTB composites (e).

The Raman spectra of the carbonized and graphitized composites based on CTP and WTB with different contents of S are shown in Figure 4. Figure 4a shows relatively high levels of structural disorder (revealed by the high intensity of the D band relative to the G band, i.e., the low *I*_G_/*I*_D_ values observed) for the carbonized graphite/CTP composites, with slightly higher levels of order found with increasing amounts of S incorporated into the system. After the composites were heated at 2500 °C, the Raman spectra of these composites (Figure 4b) showed a clear increase on the structural order (i.e., level of graphitization), as revealed by the increase of the intensity of the G band relative to the D band (i.e., higher *I*_G_/*I*_D_ values). The Raman spectra obtained for the carbonized WTB-based system (Figure 4c) showed higher levels of disorder than those found for the carbonized CTP composites in all the cases, independently of the amount of S, suggesting a more amorphous structure than the CTP, in agreement with a previous work.^11^ The level of graphitization of the system based on WTB was also found to increase considerably after the graphitization step (Figure 4d), similarly to what was observed for the CTP system, as revealed by the higher *I*_G_/*I*_D_ values found for the composites heated up to 2500 °C relative to those only treated at 850 °C. Similar structural changes seemed to occur for both systems, independently of the binder used, although the level of graphitization achieved was considerably higher for the CTP-based system, as shown in Figure 4e. We attribute this to the higher amounts of aromatic compounds present in the CTP relative to the WTB, which can be related to their graphizability (discussed in Section 3.1). Furthermore, increasing amounts of S led progressively to higher levels of graphitization (revealed by higher *I*_G_/*I*_D_ values) for both systems (Figure 4e), with optimal loadings found ∼3 wt %, above which there was no further improvement in the degree of graphitization. It should be noted that, in order to evaluate the effect of S on the binders, these Raman spectra have been taken on the CPT/WTB of the composites (not on the graphite incorporated into them). With the aim of confirming that there was no effect of the graphite on the binders’ graphitization degrees observed in the composites, Raman spectra of the binders/S mixtures containing 5 wt % S (with no graphite) were also recorded after carbonization and graphitization. The Raman spectra of these binders/S mixtures (SI) were found to be similar to those obtained from the binder of the studied graphite/binder/S composites (Figure 4).

#### 3.3.2 Physical Properties of the Carbonized and Graphitized Carbon–Carbon Composites

**Figure 5:**
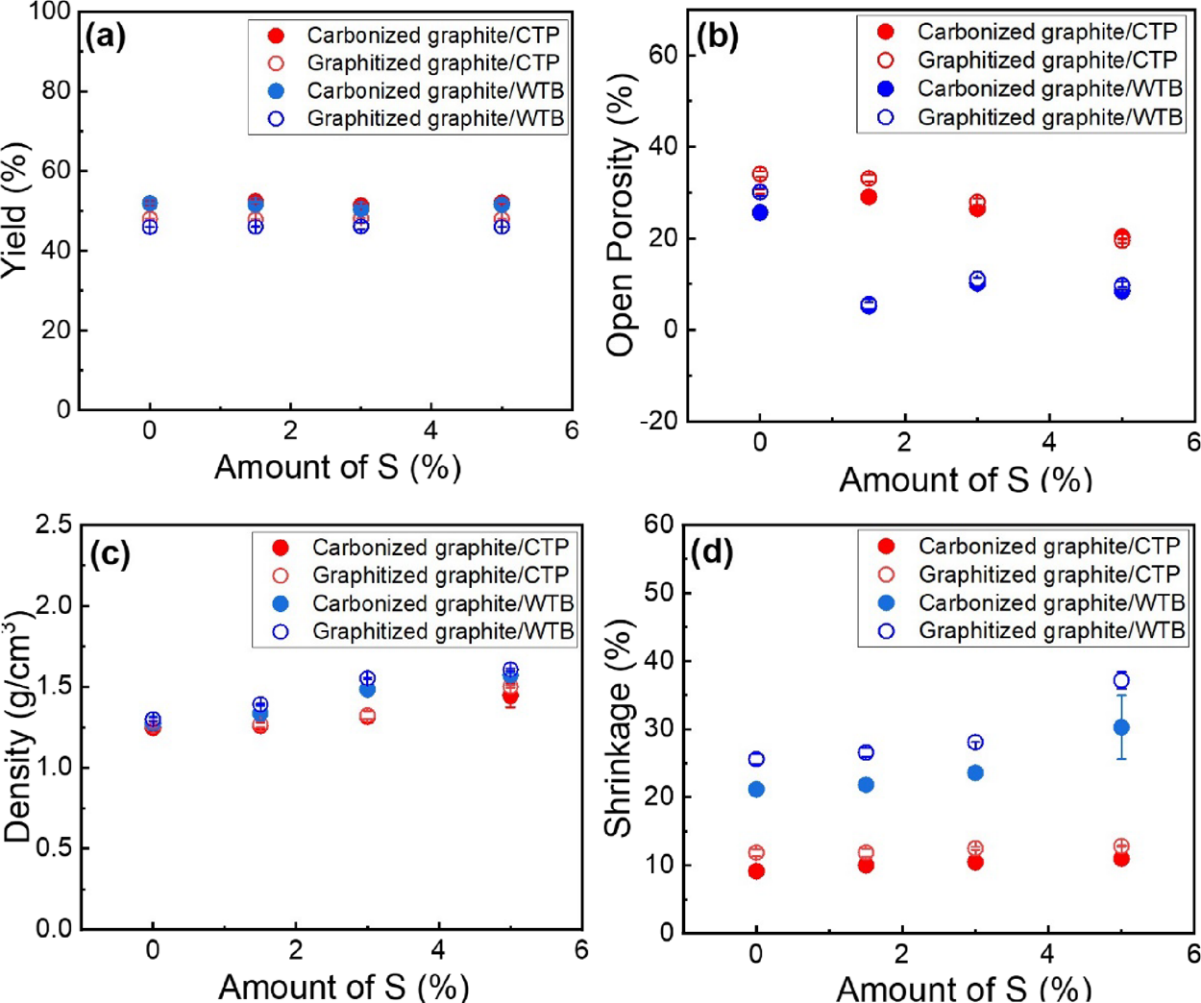
Carbon yield (%) (a), open porosity (%) (b), density (g/cm^3^) (c), and shrinkage (%) (d) of carbonized and graphitized graphite/CTP and graphite/WTB composites with different contents of S.

Figure 5a shows the variation of the carbon yield of the carbonized and graphitized composites with an increasing amount of S, as calculated using eq 3. Carbon yields for the carbonized and graphitized composites were found to be ∼52 and ∼46% for the CTP and WTB systems, respectively, regardless of the loading of S. These very similar carbon yields imply that the main mass loss occurs during carbonization, with very small amount of material being lost during the graphitization step. This observation is in agreement with both the CV and the TGA (Figure 2b) results, which showed that ∼2/3 of the binder was lost before reaching 900 °C.

Figure 5b and c shows the variation of the open porosity and apparent density, respectively, of the carbonized and graphitized composites with the amount of S. Slightly higher open porosities (thus, lower densities) were found for the CTP-based composites relative to the WTB-based composites after both carbonization and graphitization. This must be attributed to the higher amounts of volatiles with higher molecular weight found in the CTP relative to the WTB. Due to the main material loss occurring at temperatures <900 °C, the graphitized samples showed only slightly higher porosities than the carbonized ones. A progressive decrease of the open porosity (thus, increased density) with increasing amounts of S was observed for both composite systems. This suggests that this element must play a role in promoting the formation of more compact structures with less voids, probably through a cross-linking between the different particles of the composite as the volatiles are released during carbonization, in addition to the promotion of higher levels of graphitization at temperatures >850 °C (as revealed by Raman spectroscopy). It should be mentioned that these differences in open porosity found between CTP and WTB-based composites could not be identified by SEM, probably because the pores are very small and must be very well distributed in the whole composite. It is worth noting that in this work the objective was to evaluate the effect of S on the structure of the composites based on WTB and CTP, more specifically on their porosity and compactness while maintaining a 3D porous structure; hence, this discussion is focused on their apparent densities. The apparent density takes into account the pores/cavities present in the material and it directly relates to their porosity and, thus, to their overall properties. Since their real densities do not take into account the pores or cavities present in the material, the values for the real densities of the composites studied here will be higher than those found for their apparent densities and they are likely to be similar in all the cases. According to Figure 5c,d, the presence of S seems to promote the formation of more compact structures with overall lower open porosities (hence, higher apparent densities); however, the material's real density, related to the material itself, should not be affected by the presence of S.

Figure 5d shows that all the composites based on WTB exhibited higher % of shrinkage after carbonization and graphitization than those based on CTP, suggesting that the WTB must be less effective in maintaining a porous structure during thermal treatment, leading to smaller and more compact structures (as revealed by the open porosity data). Slightly higher % of shrinkage was observed for the graphitized samples than for the carbonized ones for both systems. The % of shrinkage observed in all cases, however, did not seem to be affected by the presence of S.

These results suggest that the use of WTB as a binder leads to less porous composite structures after carbonization/graphitization with higher levels of shrinkage than those based on CTP, whereas the carbon yield was very similar for both systems. In addition, the incorporation of S was found to play a significant role in the composites’ structure during carbonization and graphitization when both CTP and WTB were used as binders between the graphite particles, promoting the formation of more compact structures and increasing the level of order of the carbon-based materials during graphitization.

#### 3.3.3 Electrical Properties of the Carbon–Carbon Composites

**Figure 6:**
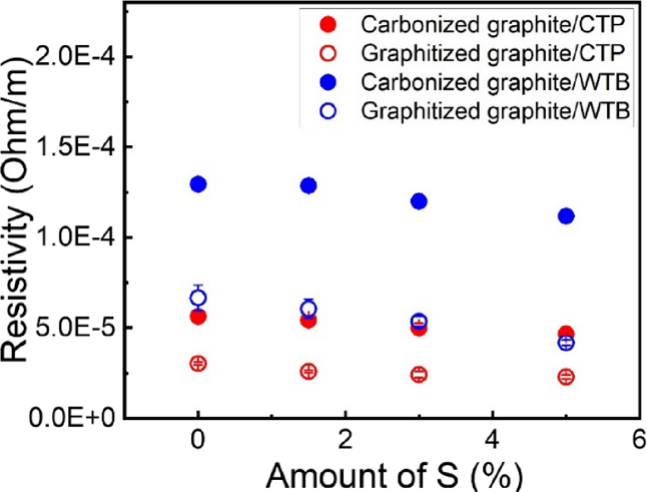
Resistivity of the carbonized and graphitized graphite/CTP/S and graphite/WTB/S composites as a function of the amount of S incorporated.

The electrical resistivities of the carbonized and graphitized composites based on CTP and WTB with different amounts of S are shown in Figure 6. The values for the graphite/WTB-based composites were found to be slightly higher than those found for the equivalent graphite/CTP composites. This must be attributed to the presence of a high number of aromatic compounds in the CTP relative to the WTB, which not only makes CTP intrinsically more conductive than WTB^19,20^ but also leads the CTP to achieve higher levels of structural order during the thermal treatments, as revealed by Raman spectroscopy (Figure 4e). (Even though the CTP composites showed higher porosities than the WTB samples, their resistivities are lower, which suggests that the level of graphitization must be playing a more important role on this property than the material's compactness) As expected, for both systems, the graphitized composites showed lower resistivities than the carbonized ones with the difference being more pronounced for the WTB system. Since the level of graphitization achieved after treating the samples at 2500 °C is considerably higher for the CTP system, this observation must be due to the more amorphous structure found for the carbonized WTB-based composites (Figure 4a,c). The electrical resistivity of both systems decreased progressively with the addition of increasing amounts of S to the composites through the promotion of more compact structures and higher levels of graphitization. Finally, it is worth noting that the electrical resistivities measured were all ∼10^–5^ Ohm/m, suggesting that any of these composites would be considered equally appropriate for conductive applications.

#### 3.3.4 Mechanical Properties of the Carbon–Carbon Composites

**Figure 7:**
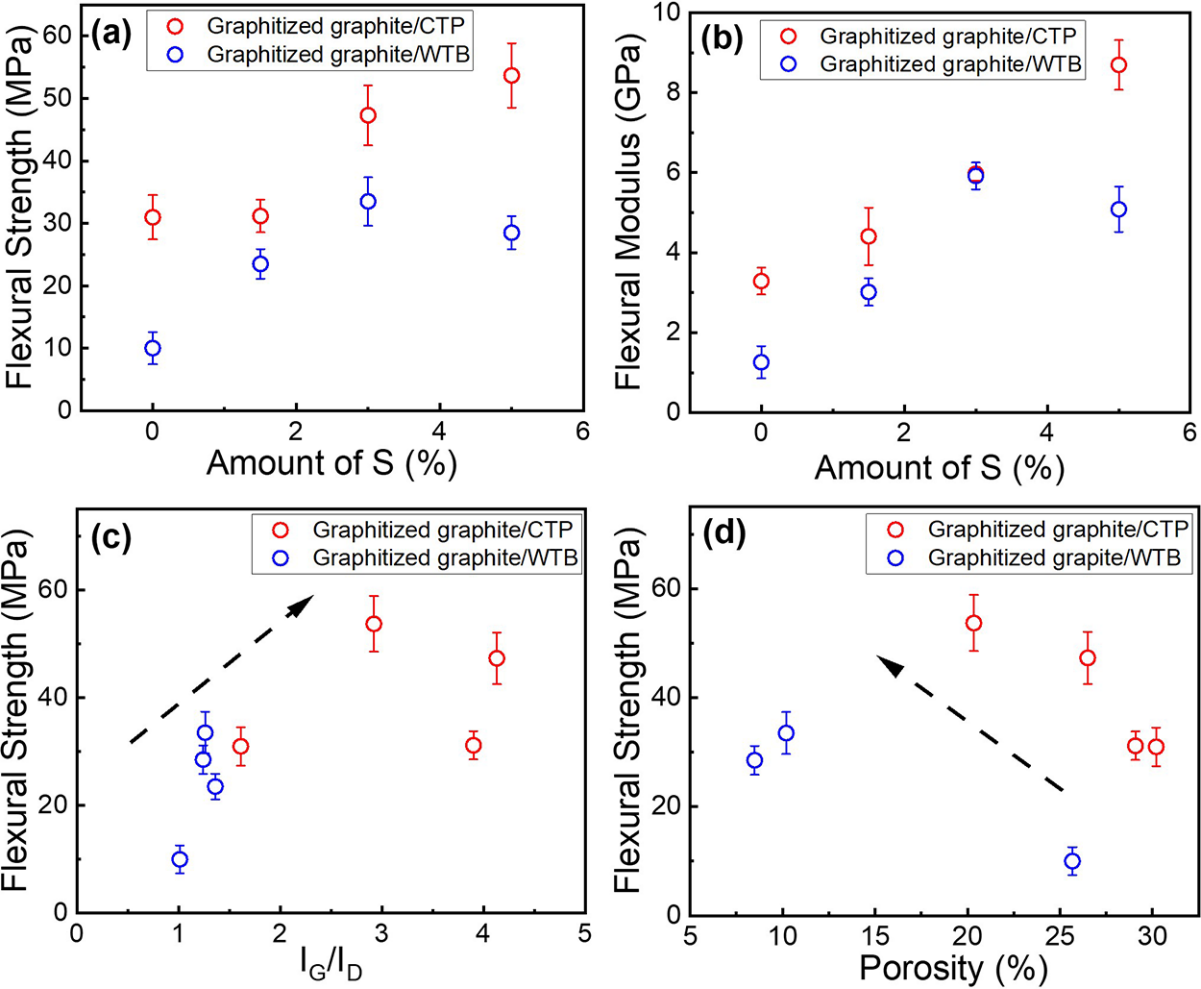
Variation of the flexural strength (a) and modulus (b) of the graphitized graphite/CTP and graphite/WTB composites with the amount of S. Variation of the flexural strength of the graphitized graphite/CTP and graphite/WTB composites with *I*_G_/*I*_D_ (c) and open porosity (d).

The mechanical properties of the graphitized composites were tested using the three-point bending method, and the variation of the flexural strength and modulus with the amount of S incorporated are shown in Figure 7a and b. Both mechanical properties were found to be superior for the composites based on CTP relative to those based on WTB, probably due to the higher levels of graphitization achieved during the thermal treatment.

Both the flexural strength and the modulus of the two series of composites were found to increase progressively with increasing amount of S, reaching maximum improvements of ∼75 and ∼233% on the flexural strength and of ∼163 and ∼354% on the flexural modulus for the WTB composite with 3 wt % loading of S (the addition of higher loadings of S did not lead to further improvements) and the CTP composite with 5 wt % loading of S (the highest loading of S studied here), respectively. The fact that the optimal S loading for the WTB system was found at 3 wt % whereas for the CTP system the mechanical properties were found to continue improving above that loading can be attributed to the lower porosities, higher densities, and higher levels of shrinkage observed for the composites based on WTB relative to those containing CTP already without S, making the effect of S on such properties considerably less pronounced for the WTB system (hence being the level of graphitization the dominant parameter for this system).

In any case, the content of S added to the composites was found to give overall lower open porosities (Figure 5b) and higher levels of graphitization (Figure 4). Thus, in order to evaluate which of these structural features is actually promoting the improvement on the mechanical properties of the graphitized composites, and in particular the strength (which is the most relevant property in industry), its variation with increasing degree of graphitization and open porosity was evaluated; the data are plotted in Figure 7c and d, respectively. Figure 7c shows that higher degrees of graphitization lead progressively to higher strengths while the opposite trend was observed for the composite porosities (Figure 7d). Typically, the porosity of these carbon–carbon composites is required to be kept within a particular range for some applications (e.g., for metal infiltration), which suggests that strategies to increase the levels of graphitization of the composites based on WTB should be developed if further improvements in their mechanical performance are required. Even though, in general, the values found for the flexural strength and moduli were lower when WTB was used as binder in these model carbon–carbon composites relative to when CTP was employed, the obtained values were found to be close enough to each other for WTB to be considered as a potential replacement for CTP, suggesting that both systems meet the mechanical requirements for industrial applications, e.g., for electric brushes.

#### 3.3.5 Role of S as a Promoter on Carbon–Carbon Composites Based on WTB and CTP

The incorporation of S as a promoter on CTP- and WTB-based composites was found to give improvements on their electrical and mechanical properties through the promotion of structural changes during both carbonization and graphitization. S was found to promote a reorganization and maybe also a cross-linking between the different particles forming the composites while chemical compounds and volatiles are being thermally released from their structure during carbonization and graphitization, leading to more compact carbon composite materials (with lower porosities and higher densities). In addition, the incorporation of S to these systems was also found to promote a further reorganization/graphitization during the graphitization step at 2500 °C, leading to highly ordered carbon–carbon composites (showing high *I*_G_/*I*_D_ values). These structural changes promoted by the S (i.e., more compact and highly graphitized composites) were probed to contribute to an improvement of the composite’s electrical and mechanical properties. TGA of the composites based on CTP and WTB containing different amounts of S (SI) revealed, however, that the presence of S did not have an effect on the non oxidative thermal degradation behavior of the composites (neither on the thermal degradation temperature nor on the residual mass remaining at 1000 °C) based on WTB or CTP, revealing that the thermal stability of these composites is not influenced by variations on their compactness, porosity, or graphitization degree. It is worth highlighting that S was found to have an important effect on the electrical and mechanical properties of the systems based on both binders; however, this effect was stronger for the CTP one relative to the one based on WTB. Even though the role of S on carbon–carbon composites based on CTP has been widely investigated in the past, the mechanism through which this element promotes structural changes is still unknown. In order to get insight into this mechanism, the elemental composition of the green, carbonized, and graphitized composites based on CTP and WTB fabricated here was analyzed by ICPS. The obtained results (SI) revealed the presence of S in the green composites (in amounts which are consistent with those incorporated in the systems during the composite fabrication), whereas an absence of this element was found in the carbonized and graphitized composites. Since the vaporization temperature of elemental S is ∼444 °C, it must be completely evaporated from the systems during the carbonization step, which suggests that it must implement some key initial structural changes on the composites before its vaporization temperature is reached, which must then derive into further structural transformations taking place as the temperature is increased, reaching highly ordered structures at 2500 °C. How those structural changes occur at temperatures above the vaporization temperature of S still remains unanswered. Further investigation is needed to understand the mechanism through which S promotes those structural changes in the composites. This knowledge will allow us to optimize the process and the composites final properties.

## 4 Conclusions

In this work, bio-oil was successfully synthesized from the pyrolysis of fresh eucalyptus sawdust, and WTB was subsequently produced from bio-oil, following a second distillation process. Results from FTIR spectroscopy and the IPA method revealed the presence of higher amounts of aromatic compounds and PAHs in the industrially used CTP relative to the WTB synthesized here, evidencing that the biopitch represents a sustainable alternative to the CTP binders currently used in industry. Green graphite/WTB and graphite/CTP composites were fabricated using methods typically used in industry, where different amounts of S were incorporated as a graphitization promoter, and they were then carbonized at 850 °C and subsequently graphitized at 2500 °C. SEM of these composites suggests a good impregnation and wetting of the graphite particles in all the cases, independent of the binder. The use of WTB as binder led to less porous composites after carbonization/graphitization with higher levels of shrinkage than those based on CTP, whereas the carbon yield was very similar for both systems. The incorporation of S was found to have a strong effect on the composites’ structure during both carbonization and graphitization independently of the binder used, promoting the formation of more compact structures and increasing their levels of graphitization. Raman spectroscopy revealed that the addition of increasing amounts of S as a promoter to the systems led progressively to higher levels of graphitization in both systems, with this effect being considerably more pronounced for the CTP system, thus showing superior electrical and mechanical properties relative to the WTB system. Even with lower levels of graphitization, the electrical and mechanical properties rendered by the WTB composites were close to those found for the CTP-based ones, suggesting that the WTB emerges as a promising competitor and potential alternative to CTP binders. In order to improve further the properties of the WTB-based composites, strategies to increase graphitization during carbonization/graphitization processes need now to be developed. Further investigation is also needed to understand the mechanism through which S promotes the observed structural changes in the composites. Understanding this mechanism will help us to design new strategies that will allow superior structural improvements of the WTB and, thus, better properties of the composites based on this alternative binder.

## Supporting Information Available

TGA and FTIR spectra of the synthesized bio-oil; chemical composition of the WTB and CTP revealed by FTIR; qualitative and quantitative evaluation of the PAHs present in CTP and WTB; SEM images of the composite cross section; Raman spectra of the (binder/S) powder mixtures; non-oxidative thermal degradation behavior of the composites; and ICPS of the composites (PDF)
