# Supplementary material for: Effect of Sulfur on Wood Tar Biopitch as a Sustainable Replacement for Coal Tar Pitch Binders
Source: ACS Appl Eng Mater. 2023 Oct 6;1(10):2567–76. doi: 10.1021/acsaenm.3c00361 (PMC10616805; doi:10.1021/acsaenm.3c00361)
Supplement: Supplementary file 1 — em3c00361_si_001.pdf [file em3c00361_si_001.pdf]

Effect of Sulphur on Wood Tar Bio-Pitch as a Sustainable Replacement for Coal Tar Pitch Binders

Zeban Shah[†]*, Mohammadhossein Saberian*[†]*, Darren Hodgeman*[‡]*, Ian Kinloch*[†] *and Cristina Vallés*[†]*

[†] Department of Materials, National Graphene Institute and Henry Royce Institute, University of Manchester, Oxford Road, Manchester, M13 9PL, UK

[‡] Carbon Science Center of Excellence, Morgan Advanced Materials and Technology, Inc., 310 Innovation Boulevard, Technology Center, Suite 250, University Park, PA 16803, USA

*Corresponding author: cristina.valles@manchester.ac.uk

SUPPORTING INFORMATION

TGA analysis and FT-IR of the synthesized bio-oil

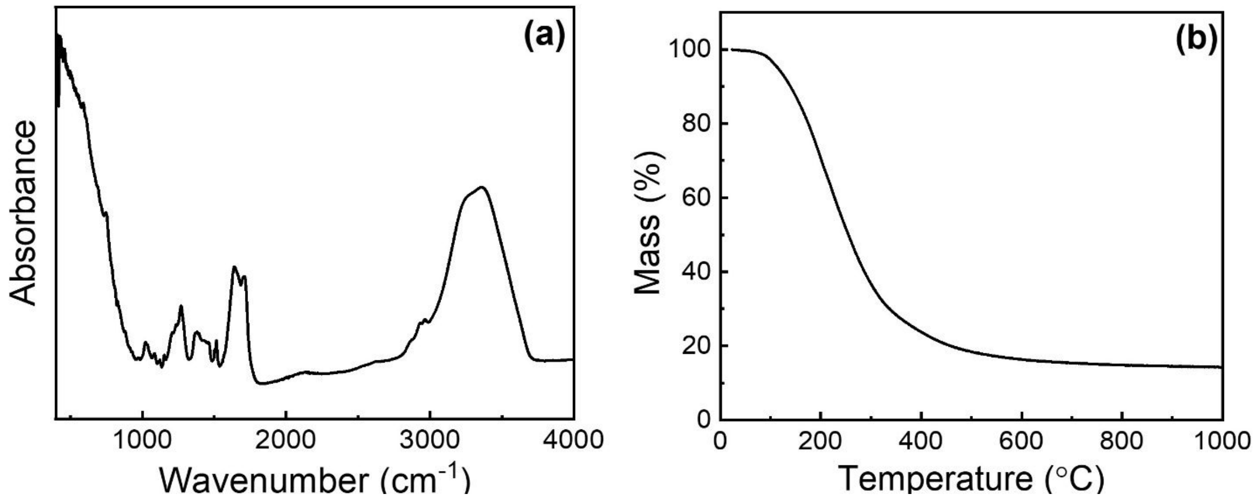

Figure S1. FT-IR (a) and TGA (b) of the bio-oil synthesized from pyrolysis of fresh eucalyptus sawdust.

Chemical composition of the WTB and CTP revealed by FT-IR

Table S1. Summary of the chemical composition of the WTB and CTP revealed by FT-IR.

**Band position (cm^{-1})**	**Associated functionalities**	**Intensity of the bands**	
		**WTB**	**CTP**
3300-3600	Moisture; hydrogen bonds, amines; C−OH stretch of −COOH	high	low
2835−2940	CH_2 and CH_3 stretch in aliphatic groups	high	medium
1700-1740	C=O containing chemical compounds (C=O stretch)	medium	--

1440-1460	bending of CH_2 and CH_3 groups in aliphatic groups	medium	medium
1110-1270	asymmetric C−O−C stretching of aliphatic ethers; C−O stretching, C−C/C=O/C−O stretching, and C−O stretching in phenols; asymmetric C−O−C stretching in alkyl aryl ethers	high	medium-low
600-860	C-H stretching of aromatic groups	--	high

Qualitative and quantitative evaluation of the PAHs present in CTP and WTB

Table S2. PAHs content for CTP and WTB determined by the EPA Method 8270D.

	PAH	Conc. (µg/kg)	
		**CTP**	**WTB**
**1**	Naphthalene	72900	N.D.<200
**2**	2-Methylnaphthalene	82500	892
**3**	1-Methylnaphthalene	37300	814
**4**	Acenaphthylene	N.D.<200	N.D.<200
**5**	Acenaphthene	608000	N.D.<200
**6**	Fluorene	275000	2850
**7**	Phenanthrene	420000	7050
**8**	Anthracene	59300	1990
**9**	Fluoranthene	1790000	2520
**10**	Pyrene	1567000	3080
**11**	Benz[a]anthracene	1610000	3000
**12**	Chrysene	972000	2220
**13**	Benzo[b]fluoranthene	1220000	8650
**14**	Benzo[j]fluoranthene	N.D.<200	N.D.<200
**15**	Benzo[k]fluoranthene	1220000	164
**16**	Benzo[a]pyrene	N.D.<200	N.D.<200
**17**	Benzo[e]pyrene	792000	2470
**18**	Benz[e]acephenanthrylene	1420000	3530
**19**	Indeno[1,2,3-cd]pyrene	683000	2320
**20**	Dibenz[a,h]anthracene	N.D.<200	N.D.<200
**21**	Benzo[ghi]perylene	189000	473
**22**	1-1'-Biphenyl	N.D.<200	N.D.<200
**23**	Carbazole	93000	N.D.<200
**24**	Dibenzofuran	37900	N.D.<200

N.D. <200 – None Detected, less than 200 parts per billion or µg/kg.

SEM images of the composites cross-section

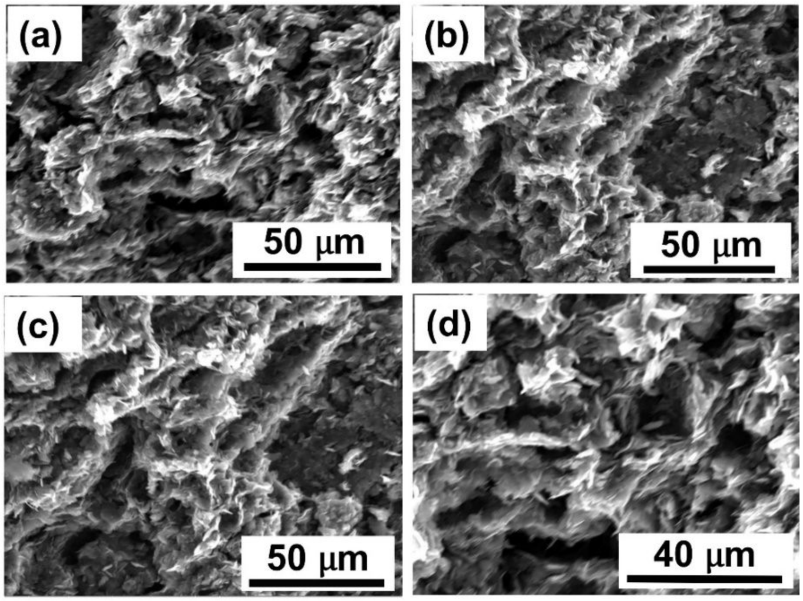

Figure S2. SEM images of the cross-section of graphitized graphite/WTB (a), graphite/WTB/S (b), graphite/CTP (c) and graphite/CTP/S (d) composites.

Raman spectra of the (binder/S) powder mixtures

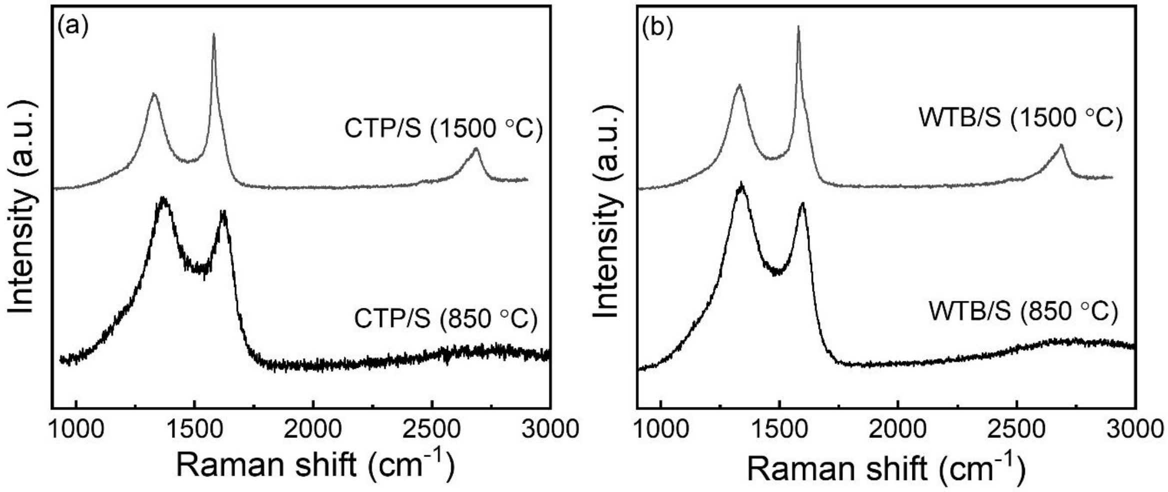

Figure S3. Raman spectra of the CTP/S and WTB/S powder mixtures after thermal treatment at 850 °C and 1500 °C.

Non-oxidative thermal degradation behaviour of the composites

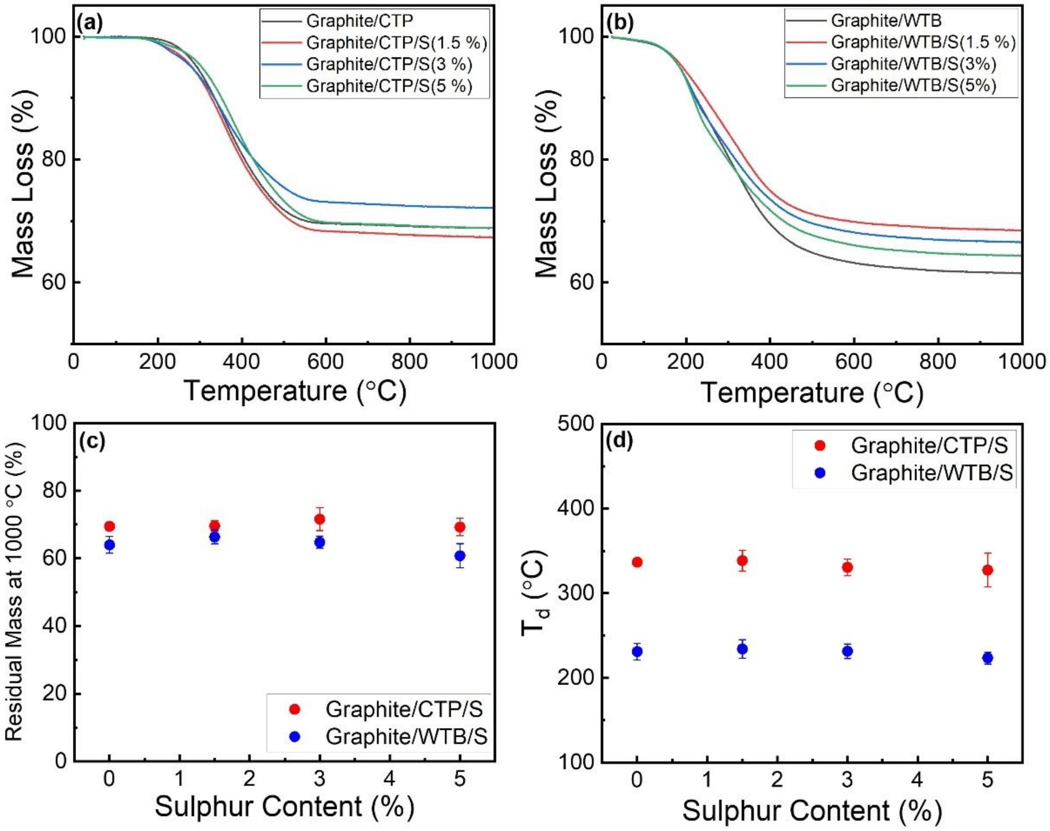

Figure S4. TGA analysis of the green Graphite/CTP/S and Graphite/WTB/S composites containing different amounts of S.

ICPS of the composites

Table S3. Content of S (wt.%) in the green, carbonized and graphitized composites determined by ICPS.

Composite	Green	Carbonized	Graphitized
Graphite/CTP/S(1.5 wt.%)	1.45	0	0
Graphite/CTP/S(3% wt.%)	2.97	0	0
Graphite/CTP/S(5 wt.%)	4.98	0	0
Graphite/WTB/S(1.5 wt.%)	1.37	0	0
Graphite/WTB/S(3 wt.%)	2.88	0	0
Graphite/WTB/S(5 wt.%)	4.28	0	0
